# Do the Successive Waves of SARS-CoV-2, Vaccination Status and Place of Infection Influence the Clinical Picture and COVID-19 Severity among Patients with Persistent Clinical Symptoms? The Retrospective Study of Patients from the STOP-COVID Registry of the PoLoCOV-Study

**DOI:** 10.3390/jpm12050706

**Published:** 2022-04-28

**Authors:** Michał Chudzik, Mateusz Babicki, Joanna Kapusta, Damian Kołat, Żaneta Kałuzińska, Agnieszka Mastalerz-Migas, Piotr Jankowski

**Affiliations:** 1 Department of Internal Medicine and Geriatric Cardiology, Medical Centre for Postgraduate Education, 01-813 Warsaw, Poland; michalchudzik@wp.pl (M.C.); piotrjankowski@interia.pl (P.J.); 2 Department of Family Medicine, Wroclaw Medical University, 51-141 Wrocław, Poland; agnieszka.mastalerz-migas@umed.wroc.pl; 3 Department of Internal Medicine and Cardiac Rehabilitation, Medical University of Lodz, 70-445 Lodz, Poland; joanna.kapusta@umed.lodz.pl; 4 Boruta Medical Center, 95-100 Zgierz, Poland; dikejs71@gmail.com (D.K.); zkkz16@gmail.com (Ż.K.)

**Keywords:** SARS-CoV-2, COVID-19, pandemic, Poland, symptoms, concomitant chronic conditions, hospitalization, home isolation

## Abstract

The severity of ailments caused by SARS-CoV-2 varies and the clinical picture has already evolved during the pandemic, complicating diagnostics. In Poland, no study has been performed to assess the clinical picture of patients across the successive pandemic waves. The aim of the study was to present the characteristics of patients who present to medical center because of persistent symptoms after COVID-19, and to study differences between hospitalized/non-hospitalized, vaccinated/non-vaccinated individuals and between different waves in Poland. This is a retrospective study evaluating the clinical presentation of COVID-19 patients from the STOP-COVID registry of the PoLoCOV-Study. This registry includes patients who present to the medical center because of persistent clinical symptoms after the isolation. The patients’ data were obtained from individuals who suffered from COVID-19 between September 2020 and December 2021.The patients were divided into groups according to the infection rate increase pattern (II/III/IV pandemic wave), status of vaccination and place of isolation. Regardless of the pandemic wave, the patients’ most commonly reported weaknesses were a cough and a headache. The arterial hypertension and hyperlipidemia were the most frequent concomitant chronic conditions. Hospitalized patients more often reported weakness or a cough while home-isolated patients were more likely to have rhinitis or a headache. Patients who completed the vaccination course showed a shorter duration of clinical symptoms and a lower mean number of symptoms. Additionally, vaccinated individuals reported less taste and/or olfactory dysfunction than unvaccinated individuals. To conclude, the persistence of the pandemic has resulted in significant changes observed in the clinical picture. Successive waves caused deterioration in the subjective assessment of the disease severity. A cough seemed to occur more frequently in the later pandemic waves.

## 1. Introduction

COVID-19, the disease caused by SARS-CoV-2, has caused the pandemic that was declared in March 2020 [1]. The disease may present varied clinical courses with symptoms associated not only with the respiratory system but also gastrointestinal system, nervous system and the sensory organs. This diversity stems from the virus biology and its ability to bind to ACE-2 receptors, which constitute the gateway into the cell. These receptors are commonly present in many tissues and organs, including the heart, intestines and liver [2,3]. The severity of individual ailments is also varied, the clinical picture may range from asymptomatic disease to rapidly progressing respiratory failure [4]. The knowledge regarding the clinical picture has significantly evolved during the pandemic. Initially, it was thought that the typical COVID-19 symptoms are fever, a dry cough and dyspnea, which constituted the clinical criteria for COVID-19 diagnosis for a very long time [5]. Additionally, smell and/or taste disorders were also included among characteristic symptoms of COVID-19 [6]. However, as the pandemic persisted, it was observed that once typical symptoms were no longer the most common manifestations of COVID-19 and were superseded by upper respiratory tract symptoms such as: sinusitis, pharyngitis, or rhinitis [7]. There were also more and more reports about gastrointestinal and cardiovascular symptoms as well as hearing disorders [6,8,9,10,11]. Moreover, the response to the disease is multifactorial and depends, amongst other things, on individual predispositions, general health status, lifestyle, as well as the virus variant and the administered vaccinations [12,13,14,15,16].

In line with the expectations of scientists all over the world, as the virus was spreading, mutations occurred within its genetic material that had direct impact on the viral infectivity, virulence, and clinical manifestations [17,18]. Next, on the turn of 2020 and 2021, the SARS-CoV-2 vaccination program was initiated in Poland [19]. Depending on the product used, the reduction in the risk of death or severe COVID-19 was demonstrated. It was also observed that the disease symptoms were much milder in vaccinated people [20]. Thus, the clinical picture of COVID-19 significantly evolved during the pandemic, involving an increasing scope of symptoms, which greatly complicated the diagnostics. To the authors’ best knowledge, no study has been performed in Poland to assess the clinical picture in COVID-19 patients across the successive waves of the pandemic. The aim of the study was to present characteristics of patients who present to medical center because of persistent symptoms after COVID-19, and to study differences between hospitalized/non-hospitalized and vaccinated/non-vaccinated individuals, and between different waves in Poland.

Analysing the previous reports, the authors formed the following research hypothesis: the clinical picture of COVID-19 differs depending on the pandemic wave and vaccination status.

## 2. Methods

### 2.1. Patients and Eligibility Criteria

This is a retrospective study evaluating the clinical presentation of COVID-19 patients from the STOP-COVID registry of the PoLoCOV-Study (ClinicalTrials.gov identifier–NCT05018052). This registry includes patients who present to the medical centre because of persistent clinical symptoms after the isolation. All of the subjects from individual groups were informed about the research assumptions and gave their written consent to participate in it.

The decisive criteria for including patients in the study were:

- SARS-CoV-2 virus infection (asymptomatic, mild, moderate, and severe course, hospitalization) confirmed by the RT-PCR test result;
- age ≥ 18;
- consent of the respondent to participate in the study;
- there are no contraindications to participate in the study.

The program inclusion criteria were uniform throughout the data collection period.

There was no exclusion criterion.

The patients’ medical data was collected on the basis of the STOP-COVID registry survey during the patient’s direct visit to the medical centre. The registry included both patients after hospitalisation and after home isolation.

Data was gathered from patients who suffered from COVID-19 in the period from September 2020 to December 2021. The patients were divided into three groups according to the infection rate increase pattern in Poland (acquired from Worldometer website at https://www.worldometers.info/coronavirus/country/poland, accessed on 25 April 2022). The criterion for belonging to the group was the period of isolation. Due to a small number of first wave patients (cases from 1 March 2020 to 30 August 2020), they were not included in the final analysis. The aforementioned three groups were:

- Group 1 (II wave) cases–from 1 September 2020 to 30 January 2021;
- Group 2 (III wave) cases–from 1 February 2021 to 30 August 2021;
- Group 3 (IV wave) cases–from 1 September 2021 to the end of the follow-up period.

At each visit, the patient was asked to fill in a questionnaire regarding their health status when suffering from COVID-19, including basic socio-demographic data such as age, gender, body mass and height (used to calculate BMI), medical history concerning chronic conditions and medications. Additionally, information was collected on the course of COVID-19, including the date of onset of symptoms, place of isolation, duration of symptoms and subjective assessment of disease severity on a scale from 0 to 3 (0—asymptomatic, 1—mild, 2—moderate, 3—severe COVID-19). The questionnaire comprised the most common clinical symptoms of COVID-19, such as: fever, subfebrile states, chills, a cough, shortness of breath, smell/taste/hearing disorders, headaches, myalgia, arthralgia, chest pain. In addition, vaccination status was assessed among the patients of the 3rd and 4th wave of the pandemic. During this period, vaccinations against COVID-19 were widely available in Poland, therefore the scope of collected data was extended. A vaccinated person is one who has completed the full immunization schedule.

The next part of the questionnaire concerned the health state assessment at the time of the visit, including physical examination. 

The criteria for assessing the severity of COVID-19 were as follows:

1 Asymptomatic:
  - no symptoms;
  - parainfluenza symptoms of upper respiratory tract infection for up to 3 days.

2 Mild:
  - home course of infection;
  - subjective evaluation by the patient as a light course (“1” on a 1–3 scale);
  - duration of symptoms up to 7 days.

3 Moderate:
  - subjective evaluation by the patient as moderate/severe course (“2 or 3” on a 1–3 scale);
  - duration of symptoms more than 7 days but less than 14 days;
  - temperature > 38 °C and dyspnea

4 Severe:
  - one of the following: hospitalization with diagnosis: pneumonia, respiratory failure, intensive care unit, assisted breathing, thromboembolic complications during hospitalization or;
  - home course with symptoms lasting >14 days, subjective evaluation by the patient as severe (“3” on a scale of 1–3), with temperature >38 °C, dyspnea or saturation below 94 lasting more than 3 days.

Subsequently, the patient received recommendations regarding further diagnostics and/or treatment and follow-up visit schedule. The visits were scheduled at 3 months and 12 months after the initial visit.

The study was carried out in conformance with the Declaration of Helsinki and approved by the Bioethics Committee of the Medical University of Wrocław.

### 2.2. Statistical Analysis

The statistical analysis was performed using Statistica software from StatSoft. Qualitative and quantitative variables were analyzed. The chi-square test was used to determine the relationship between the compared ordinal variables. Depending on the number, Chi-squared test with Yates’s correction for continuity and Fisher’s exact test were used. In the case of quantitative variables, basic descriptive statistics were used. The statistical significance of differences between mean values was assessed using the nonparametric Mann–Whitney *U* Test.

In every case, the adopted statistical significance level was <0.05.

## 3. Results

### 3.1. Study Group Characteristics

The precise summary of patients included in the analysis is presented in Table 1, including both the entire group and the successive pandemic waves. Up to now, data has been collected from 2300 patients, but 1961 convalescent patients were included in the final analysis. A total of 339 patients were rejected from the analysis because of incomplete data. The vast majority were women, 1219 (62.2%), and the mean age was 52.3 years (min. 16, max. 92, standard deviation (SD) 13.69). 1069 (47.8%) patients contracted the disease during the second wave of the COVID-19 pandemic in Poland, 782 (34.9%) during the third wave, and 110 (4.9%) during the fourth wave. In the entire group, 1269 subjects (66.1%) had at least 1 chronic condition. Among them, the most common condition was hypertension, present in 742 patients (37.8%) and hyperlipidemia in 414 (21.1%) cases. A higher proportion of hospitalized patients were males (50.2%) and suffered from hypertension (54.7%) and diabetes (18.4%). Hospitalized patients also had higher mean age and BMI than those in home isolation. In the group of patients from waves 3 and 4 of the pandemic, 184 (20.6%) became ill after a course of COVID-19 vaccination. Men were more likely to be vaccinated in the study group. There were no differences in age, anthropometric measurements, or chronic conditions. Those vaccinated against COVID-19 were also more likely to have been vaccinated against influenza in the previous season. Vaccinated individuals were less likely to be hospitalized for COVID-19 (78.4% vs. 84.3%).

### 3.2. COVID-19 Clinical Symptom Characteristics

**Figure 1:**
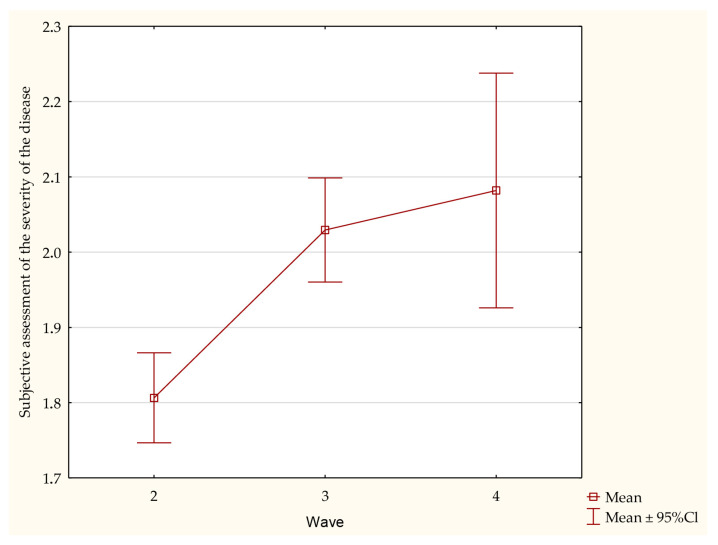
Comparison of the subjective assessment of SARS-CoV-2 infection severity in reference to the respective stages of the study.

**Figure 2:**
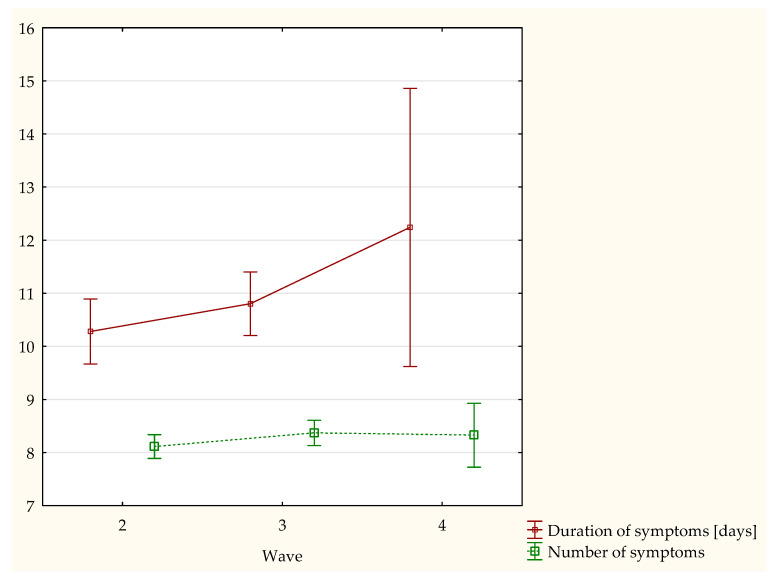
Comparison of the time of symptom duration and number of symptoms in reference to the respective stages of the study.

Among all of the studied patients, 1949 (99.4%) declared the occurrence of at least 1 clinical symptom of COVID-19. The mean number of symptoms was 8.22 ± 3.7, with the highest mean in the third wave of the pandemic: 8.34 ± 3.40. The most frequently occurring clinical symptom was weakness: 1502 (76.6%) patients. In subsequent waves, there was a growing trend towards patients declaring the occurrence of rhinitis or a cough. In the subjective assessment of COVID-19 severity, the patients most often selected the highest value (3) in all the analyzed waves. Moreover, it was demonstrated that with every subsequent wave, the subjective impression of disease severity was increasing (*p* < 0.001) (Figure 1). On the other hand, no statistically significant differences were demonstrated in either the number or duration of clinical symptoms between the respective stages of the study (Figure 2). The precise summary of the clinical symptoms in the entire study population and successive pandemic waves are presented in Table 2.

### 3.3. Summary of the Clinical Symptoms in Patients Subjected to Home Isolation and Hospitalisation

In a comparative analysis of home-isolated and hospitalized patients, it was demonstrated that home isolation was more frequently associated with normal body temperature or subfebrile states, rhinitis, and a headache (*p* < 0.001). The patients who experienced COVID-19 at home had smell and taste disorders statistically more frequently than hospitalized patients. In the hospitalized patients, severe weakness (*p* = 0.024), fever, a cough and dyspnea (*p* < 0.001) were more prevalent. Interestingly, no differences were observed with regard to chest pain. In the subjective assessment of disease severity, hospitalized patients had on average 0.7-point higher values on the 4-grade scale compared to patient treated at home. The precise comparison of the clinical symptoms is presented in Table 3.

### 3.4. The Impact of Vaccination on COVID-19 Clinical Manifestations

Analysis of the collected data showed that vaccinated individuals were less likely to be hospitalized for COVID-19 (78.4% vs. 84.3% *p* = 0.050 (Table 1)). However, when comparing the clinical presentation of COVID-19, it was observed that vaccinated subjects were less likely to report headaches, back pain, or leg pain. The vaccinated individuals reported less taste and/or olfactory dysfunction than unvaccinated individuals (28.7% vs. 45.7%; *p* < 0.001). There was no difference in subjective assessment of disease severity or dyspnea or chest pain. In addition, patients after vaccination had a shorter duration of clinical symptoms (*p* = 0.008) and a lower average number of symptoms (*p* = 0.013). A detailed summary of the results is shown in Table 4.

## 4. Discussion

The aim of this study was to present the main clinical symptoms occurring in COVID-19 patients from the perspective of the successive waves of the pandemic in Poland, as well as to compare patients subjected to home isolation and hospitalization.

Of the 1961 analyzed clinical cases, 1949 reported the occurrence of at least 1 disease symptom, with the mean number of 8.22 ± 3.6 and the mean duration of 10.6 ± 9.4 days. Regardless of the pandemic wave, among all analyzed symptoms, the patients most commonly reported significant weakness (76.6%), a cough (65.3%) and a headache (57.8%). Smell and/or taste disorders occurred in every second patient (53.8%). Febrile symptoms were reported by 53.7% of patients.

With regards to the concomitant chronic conditions, arterial hypertension and hyperlipidemia were most frequently reported, both in the successive waves and in the collective assessment. In the case of hypertension, this confirms the data acquired in a meta-analysis of patients with severe COVID-19 and those admitted to the intensive care unit [21].

An interesting aspect is the way the patients subjectively assessed the severity of the disease (at a scale from 0 to 3) in successive pandemic waves. According to their subjective assessment, the disease was mostly asymptomatic or mild in the second wave, while a moderate and severe course was most often reported in the fourth and third wave of the pandemic. This suggests that the subjective assessment deteriorated after the second wave; therefore, it is possible to divide the scale according to the waves: 0–1 (second wave) and 2–3 (third and fourth wave). Since many studies do not differentiate between the pandemic waves (which constitutes a significant advantage of our study), it is difficult to identify differences between individual waves in literature; additionally, with time, more and more studies appear regarding the earlier stages of the pandemic. A study by Chams et al. demonstrated that the most common symptoms of COVID-19 include fever, a cough, weakness and dyspnea [22]—considering the frequency of symptom occurrence in our patients, fever or dyspnea are not the most common symptoms, while a cough or weakness (in variable order) were very often the two most common symptoms. A cough as the most common symptom would be consistent with the multicentre study by Bhatraju et al. [23] or a meta-analysis by Jain & Yuan [21]. A clinical evaluation by Guan et al., has also demonstrated that a cough is a common symptom, being the second most prevalent [24]. Similar results were obtained in a meta-analysis by Gómez-Ochoa, who assessed symptoms in health care professionals [25]. In the aforementioned studies, weakness was often the next most prevalent symptom after a cough, thus we decided to use the opportunity provided by our study to check whether the frequency of these symptoms does not change depending on the wave. When analyzing all of the symptomatic cases, we observed that the order of symptoms according to the frequency of their occurrence was most often the following: weakness, a cough, a headache, and back pain. In the second wave, however, weakness preceded smell and/or taste disorders, which were followed by cough and headache. Third wave patients also reported weakness most often, which was followed by a cough, a headache, and fever. Interestingly, a cough occurred more frequently than weakness in fourth wave patients, and the third most prevalent symptoms were, ex aequo, headache, fever, as well as smell and/or taste disorders. We may conclude that, considering symptoms by wave, the frequency of a cough gradually increased from wave to wave, eventually surpassing even the prevalence of weakness.

In this study, we have also compared the clinical symptoms in patients during home isolation and hospitalization. Among the statistically significant results, hospitalized patients more often reported weakness, a cough, fever or dyspnea. In contrast, patients staying at home were more likely to have a temperature < 36.6 or between 36.6 and 37.5, rhinitis, a headache, or symptoms associated with smell and/or taste disorders (3 independent parameters). The latter results are consistent with a meta-analysis in which anosmia and ageusia were predominant among non-hospitalized patients [26]. The same authors, using additional data from another meta-analysis [27], indicated that weakness, a cough, fever and dyspnea were among the most common symptoms in hospitalized patients. Another interesting aspect seems to be the higher prevalence of symptoms related to smell or taste in patients in home isolation. The authors have speculated that this could be related to the increased use of steroids. Both nasal and oral corticosteroids were used to treat patients with smell disorders, however, they were administered in a relatively small percentage (10.6%) of patients, as reported in a multi-centre study by Lechien et al. [11]. A case report by Touisserkani & Ayatollahi confirmed the efficiency of treating the loss of smell with oral prednisolone [28]. However, a randomized controlled trial did not recommend the use of nasal betamethasone to facilitate recovery from acute anosmia [29]. Further research regarding corticotherapy is necessary.

Moreover, in a large analysis involving more than 33,000 patients in Japan, some of the most commonly reported symptoms on admission were fever and a cough, which is consistent with our observations. These symptoms, along with dyspnea, are associated with a higher risk of hospitalization [30,31].

In addition, our observation compared the clinical presentation of patients in relation to COVID-19 vaccination. The analysis of individual clinical symptoms showed that vaccinated subjects reported leg pain, headache, or back pain less frequently. Moreover, the vaccinated individuals reported less taste and/or olfactory dysfunction than unvaccinated individuals. The literature lacks data assessing the effect of vaccination status on the incidence of olfactory and/or taste disorders. For example, the observation by Vaira, L. et al., shows that vaccination does not reduce the incidence of these symptoms [32]. Avci, H. et al., made similar observations [33]. These data are inconsistent with our results, which, combined with the small amount of data, indicates the need for further exploration of the topic, especially since these symptoms are one of the most characteristics of COVID-19 [6]. They were also more likely to be isolated at home, which seems to confirm the effectiveness of vaccination in reducing the risk of hospitalization, confirming previous reports [34,35].

The authors are aware of the limitations of this study, which undoubtedly comprises the lack of knowledge concerning the use of pharmacotherapy in the course of the disease. Although clear guidelines for the treatment of COVID-19 were provided during the pandemic, physicians often invented their own regimens, which included antibiotic therapy and antiviral drugs, amantadine or ivermectin. According to global reports, these drugs do not affect the course of the disease and do not lower the risk of its severity but the heterogeneity in their use could have affected the course of the disease in individual patients. Neither did we know the SARS-CoV-2 variant with which the individual patients were infected; this aspect undoubtedly had a huge impact on the clinical picture of the disease. The data available in Poland (acquired from https://sarswpolsce.pl/, accessed on 25 April 2022) shows that, in the study period, the most prevalent variant in Group 1 was the primary one, in Group 2 it was variant B.1.1.7 (British), and variant B.1.617.2 (delta) in Group 3.

Precise knowledge of the variants would allow us to better characterize the individual pandemic waves in Poland, as well as to present a typical clinical picture for each of them. From the available literature, we know that as a result of the ability of viruses to mutate, they acquire new characteristics, which translates into a different level of infectivity, virulence conditioning the diversity in the clinical picture, response to vaccination or sensitivity to treatment [36,37,38,39]. Careful monitoring of this phenomenon is extremely important because of the lack of knowledge on the direction of further virus mutations. Currently, there are also data on so-called viral hybrids, for example, Omicron-Delta called “deltacrone” [40].

Another limitation is the study group selection. These were people who, due to persistent symptoms after contracting COVID-19, were seeking further diagnosis and treatment. According to this review results, over 99% of patients presented clinical symptoms, which obviously contradicts current medical evidence, which indicates that COVID-19 may be asymptomatic in even 8–30% of cases [41]. Due to the above, the results of this study should not be analyzed in relation to the entire population. Another methodological limitation is the period of data collection, which took place after the disease contraction. The time delay is associated with the risk that patients might fail to report all of the symptoms.

Summing up, this study provides information on the clinical picture in patients suffering from COVID-19 in relation to successive waves of the pandemic in Poland. Despite many limitations, the collected data constitutes a significant contribution to the way we perceive the disease and shows us the variability of the clinical picture in the successive waves of the pandemic or in relation to the place of undergoing treatment. The study is still ongoing, and we plan to publish the results of longitudinal observations including the assessment of the post-COVID-19 syndrome in the near future.

## 5. Conclusions

COVID-19 is a disease characterized by a diverse clinical picture, with the most common manifestations including weakness and a cough. The persistence of the pandemic, emergence of new virus mutations and vaccination program have resulted in significant changes observed in the clinical picture of the disease. Each successive wave caused statistically significant increases in the subjective assessment of the disease severity. A cough seemed to occur more frequently in the later waves of the pandemic.

## Figures and Tables

**Table 1:** Study group characteristics with reference to successive stages of the study, place of isolation and COVID-19 vaccination status.

	Variable	The Whole Group (*n* = 1961)	Second Wave (*n* = 1069)	Third Wave (*n* = 782)	Fourth Wave (*n* = 110)	Home Insulation (*n* = 1619)	Hospitalization (*n* = 342)	Vaccinated (*n* = 184)	Unvaccinated (*n* = 708)	*p* *
Sex	Female	1219 (62.2)	655 (62.3)	489 (62.5)	75 (68.2)	1051 (65.5)	168 (40.8)	132 (28.6)	277 (39.1)	0.011
	Male	742 (37.8)	397 (37.7)	293 (37.5)	35 (31.8)	568 (35.5)	174 (50.2)	53 (71.4)	432 (60.9)	
Age (M ± SD)		52.8 ± 13.6	51.9 ± 13.3	54.3 ± 13.7	51.0 ± 14.9	51.6 ± 13.3	59.1 ± 13.1	54.04 ± 14.4	53.8 ± 13.7	0.793
Weight [kg] (M ± SD)		79.9 ± 17.8	79.6 ± 17.8	80.8 ± 18.1	76.9 ± 15.5	79.1 ± 17.6	83.8 ± 18.1	78.1 ± 16.2	80.9 ± 18.2	0.108
Height [cm] (M ± SD)		169 ± 9.1	169.7 ± 9.0	168.9 ± 9.1	168.1 ± 15.5	169.3 ± 8.9	169.1 ± 9.7	167.5 ± 9.5	169 ± 9.0	0.051
BMI (M ± SD)		27.8 ± 5.5	27.6 ± 5.5	28.2 ± 5.5	27.3 ± 5.8	27.5 ± 5.5	29.2 ± 5.4	27.8 ± 5.6	28.8 ± 5.6	0.434
Chronic diseases	Hypertension	742 (37.8)	371 (34.7)	331 (42.3)	40 (36.4)	555 (34.2)	187 (54.7)	76 (41.1)	296 (41.8)	0.924
	Diabetes	210 (10.7)	98 (9.2)	101 (12.9)	11 (10.0)	147 (9.1)	63 (18.4)	21 (11.4)	91 (12.9)	0.671
	Coronary artery disease	129 (6.6)	67 (6.3)	55 (7.0)	7 (6.4)	81 (5.0)	48 (14.0)	16 (8.7)	46 (6.5)	0.388
	Heart failure	67 (3.5)	28 (2.6)	38 (4.9)	1 (0.9)	50 (3.1)	17 (5.0)	0 (0.0)	39 (5.5)	<0.001
	Venous thromboembolism	22 (1.2)	13 (1.2)	9 (1.1)	0 (0)	18 (1.1)	4 (1.2)	3 (1.5)	6 (0.9)	0.573
	Hyperlipidemia	414 (21.1)	209 (19.6)	183 (23.4)	22 (20.0)	340 (21.0)	74 (21.6)	41 (22.2)	165 (23.3)	0.817
	Asthma	182 (9.3)	98 (9.2)	72 (9.2)	12 (10.9)	140 (8.7)	42 (12.3)	16 (8.7)	68 (9.6)	0.791
	COPD *	49 (2.5)	17 (1.6)	31 (3.9)	1 (0.9)	35 (2.2)	14 (4.1)	7 (3.8)	25 (3.5)	0.954
	Thyroid disease	326 (16.7)	176 (16.5)	127 (16.2)	21 (19.1)	269(16.5)	58 (16.5)	33 (17.8)	116 (16.2)	0.246
A place of isolation	Home	1619 (82.5)	909 (85.0)	614 (78.5)	96 (87.3)	1619 (100)	0 (0.0)	156 (84.3)	555 (78.4)	0.050
	Hospital-without pneumonia	63 (1.5)	38 (3.6)	24 (3.1)	1 (0.9)	0 (0.0)	63 (18.4)	3 (1.6)	8 (1.1)	0.868
	Hospital-with pneumonia	272 (13.9)	120 (11.2)	139 (17.8)	13 (11.8)	0 (0.0)	272 (79.5)	25 (13.5)	127 (17.9)	0.188
	Hospital-ICU	7 (0.4)	2 (0.2)	5 (0.6)	0 (0.0)	0 (0.0)	7 (2.1)	1 (0.5)	4 (0.6)	0.966
Flu vaccinations in the previous season		164 (8.4)	84 (7.9)	70 (8.9)	10 (9.3)	132 (8.2)	32 (9.5)	30 (16.3)	50 (7.1)	<0.001

COPD—chronic obstructive pulmonary disease. ICU—intensive care unit M–mean, SD—standard deviation. * *p*-value refers to comparison of vaccinated with unvaccinated patients.

**Table 2:** Clinical manifestation of COVID-19 in the entire population and in successive waves of the pandemic.

Symptom		Second Wave (*n* = 1069)	Third Wave (*n* = 782)	Fourth Wave (*n* = 110)	*p* *	The Whole Group (*n* = 1961)
Patient with clinical symptoms		1060 (99.2)	779 (99.6)	110 (100.0)	0.320	1949 (99.4)
Temperature < 36.6 °C		166 (15.5)	128 (16.4)	14 (12.8)	0.599	308 (15.7)
Temperature 36.6–37.5 °C		249 (23.3)	162 (20.7)	26 (23.6)	0.551	437 (22.3)
Temperature > 37.5 °C		554 (51.8)	439 (56.1)	61 (55.5)	0.172	1054 (53.7)
Cough		653 (61.1)	541 (69.2)	87 (79.0)	**<0.001**	1281 (65.3)
Dyspnoea		499 (46.7)	433 (55.4)	48 (43.6)	**<0.001**	980 (49.9)
Rhinitis		310 (29.0)	276 (35.3)	58 (52.7)	**<0.001**	644 (32.8)
Isolated olfactory dysfunction		118 (11.0)	50 (6.4)	16 (14.6)	**<0.001**	184 (9.4)
Isolated taste dysfunction		57 (5.3)	48 (6.1)	8 (7.3)	0.598	113 (5.8)
Taste and olfactory dysfunction		544 (50.9)	283 (36.2)	61 (55.5)	**<0.001**	888 (45.3)
Taste and/or olfactory dysfunction		679 (63.5)	349 (44.6)	27 (24.6)	**<0.001**	1055 (53.8)
Weakness		793 (74.2)	625 (79.9)	84 (76.4)	**0.015**	1502 (76.6)
Chest pain		465 (43.5)	393 (50.3)	50 (45.5)	**0.016**	908 (46.3)
Back pain		594 (55.6)	433 (55.4)	53 (48.2)	0.326	1080 (55.1)
Leg pain		484 (45.3)	374 (47.8)	48 (43.6)	0.474	906 (46.2)
Headache		614 (57.4)	458 (58.6)	61 (55.5)	0.781	1138 (57.8)
Arthralgia		449 (42.0)	375 (48.0)	44 (40.0)	**0.025**	868 (44.3)
Diarrhoea		222 (20.8)	171 (21.9)	23 (20.9)	0.845	416 (21.2)
Vomits		66 (6.2)	64 (8.2)	8 (7.3)	0.247	138 (7.0)
Chills		399 (37.3)	316 (40.4)	45 (40.9)	0.361	760 (38.8)
Labile blood pressure values		150 (14.0)	138 (17.7)	20 (18.2)	0.082	308 (15.7)
Hearing dysfunction		92 (8.6)	103 (13.2)	16 (14.6)	**0.003**	211 (10.8)
Average number of symptoms		8.11 ± 3.7	8.4 ± 3.4	8.32 ± 3.2	0.973 **	8.22 ± 3.6
Duration of symptoms [days]		10.3 ± 9.9	10.8 ± 8.1	12.2 ± 8.5	0.298 **	10.6 ± 9.4
Subjective assessment of the severity of the disease	0	105 (9.8)	64 (8.2)	0 (0.0)	**<0.001**	169 (8.6)
	1	340 (31.8)	176 (22.5)	33 (30.0)		549 (28.0)
	2	281 (26.3)	215 (27.5)	35 (31.8)		531 (27.1)
	3	343 (32.1)	327 (41.8)	42 (38.2)		712 (36.3)
	M SD	1.81 ± 0.9	2.03 ± 0.9	2.08 ± 0.8	<0.001 **	1.9 ± 0.9

* Chi-squared test. ** Mann–Whitney *U* Test. Statistically significant values are in bold with the significance level set at *p* < 0.05.

**Table 3:** Comparison of the clinical picture between hospitalized patients and patients staying in home isolation in the course of COVID-19.

Symptom	Home Insulation	Hospitalization	*p* *
Patient with clinical symptoms	1607 (99.3)	342 (100.0)	0.110
Temperature < 36.6 °C	284 (17.5)	24 (7.0)	**<0.001**
Temperature 36.6–37.5 °C	389 (24.0)	48 (14.0)	**<0.001**
Temperature > 37.5 °C	809 (49.9)	245 (71.6)	**<0.001**
Cough	1033 (63.8)	248 (72.5)	**<0.001**
Dyspnoea	730 (45.1)	250 (73.1)	**<0.001**
Rhinitis	572 (35.3)	72 (21.1)	**<0.001**
Isolated olfactory dysfunction	171 (10.5)	13 (3.8)	**<0.001**
Isolated taste dysfunction	91 (5.6)	22 (6.4)	0.558
Taste and olfactory dysfunction	772 (47.7)	116 (33.9)	**<0.001**
Taste and/or olfactory dysfunction	934 (57.7)	121 (35.4)	**<0.001**
Weakness	1224 (75.6)	278 (81.3)	**0.024**
Chest pain	751 (46.4)	157 (45.9)	0.871
Back pain	893 (55.2)	187 (54.6)	0.872
Leg pain	749 (46.3)	157 (45.9)	0.904
Headache	974 (60.2)	159 (46.5)	**<0.001**
Arthralgia	719 (44.4)	149 (43.6)	0.775
Diarrhoea	336 (20.8)	80 (23.4)	0.278
Vomits	110 (6.8)	28 (8.2)	0.360
Chills	632 (39.0)	128 (37.4)	0.578
Labile blood pressure values	244 (15.1)	64 (18.7)	0.095
Hearing dysfunction	171 (10.6)	40 (11.7)	0.548
Average number of symptoms	8.28 ± 3.6	7.98 ± 3.51	0.239 **
Duration of symptoms [days]	10.49 ± 9.3	11.05 ± 10.1	0.509 **
Subjective assessment of the severity of the disease	1.8 ± 0.9	2.50 ± 1.1	<0.001 **

* Chi-squared test. ** Mann–Whitney *U* Test. Statistically significant values are in bold with the significance level set at *p* < 0.05.

**Table 4:** Comparison of the clinical picture between vaccinated and unvaccinated patient in second and third wave.

Symptom	Vaccinated (*n* = 184)	Unvaccinated (*n* = 708)	*p* *
Patient with clinical symptoms	180 (97.3 )	694 (98.0)	0.543
Temperature < 36.6 °C	29 (15.7)	112 (15.9)	0.965
Temperature 36.6–37.5 °C	37 (19.8)	150 (21.3)	0.780
Temperature > 37.5 °C	97 (52.4)	402 (57.0)	0.300
Cough	132 (71.4)	497 (70.5)	0.891
Dyspnoea	93 (50.3)	387 (54.9)	0.298
Rhinitis	77 (41.6)	257 (36.5)	0.227
Isolated olfactory dysfunction	12 (6.5)	54 (7.7)	0.587
Isolated taste dysfunction	8 (4.3)	47 (6.7)	0.314
Taste and olfactory dysfunction	79 (42.7)	265 (37.6)	0.235
Taste and/or olfactory dysfunction	53 (28.7)	322 (45.7)	**<0.001**
Weakness	140 (75.7)	566 (80.3)	0.202
Chest pain	83 (44.9)	360 (51.1)	0.156
Back pain	85 (45.9)	401 (56.9)	**0.010**
Leg pain	72 (38.9)	349 (49.5)	**0.013**
Headache	92 (49.7)	425 (60.3)	**0.012**
Arthralgia	75 (40.5)	343 (48.7)	0.059
Diarrhoea	34 (18.4)	159 (22.6)	0.260
Vomits	16 (8.7)	56 (7.9)	0.872
Chills	78 (42.2)	282 (40.0)	0.653
Labile blood pressure values	33 (17.8)	125 (17.7)	0.940
Hearing dysfunction	27 (14.6)	91 (12.9)	0.631
Average number of symptoms	7.8 ± 3.25	8.5 ± 3.38	0.013 **
Duration of symptoms [days]	11.8 ± 11.1	12.6 ± 7.32	0.008 **
Subjective assessment of the severity of the disease	2.0 ± 0.89	2.1 ± 0.98	0.372 **

* Chi-squared test. ** Mann–Whitney *U* Test. Statistically significant values are in bold with the significance level set at *p* < 0.05.

## Data Availability

The data presented in this study are available on request from the corresponding author.
